# Effects of Probiotics and Gut Microbiota on Bone Metabolism in Chickens: A Review

**DOI:** 10.3390/metabo12101000

**Published:** 2022-10-20

**Authors:** Pan Chen, Tingting Xu, Chaodong Zhang, Xishuai Tong, Aftab Shaukat, Yanfeng He, Kaili Liu, Shucheng Huang

**Affiliations:** 1 College of Veterinary Medicine, Henan Agricultural University, Zhengzhou 450002, China; 2 Institutes of Agricultural Science and Technology Development (Joint International Research Laboratory of Agriculture and Agri-Product Safety of the Ministry of Education of China)/College of Veterinary Medicine, Yangzhou University, Yangzhou 225009, China; 3 National Center for International Research on Animal Genetics, Breeding and Reproduction (NCIRAGBR), Huazhong Agricultural University, Wuhan 430070, China

**Keywords:** bone metabolism, gut microbiota, gut–bone axis, leg disease, probiotics, poultry

## Abstract

Broiler leg diseases are a common abnormal bone metabolism issue that leads to poor leg health in growing poultry. Bone metabolism is a complicated regulatory process controlled by genetic, nutritional, feeding management, environmental, or other influencing factors. The gut microbiota constitutes the largest micro-ecosystem in animals and is closely related to many metabolic disorders, including bone disease, by affecting the absorption of nutrients and the barrier function of the gastrointestinal tract and regulating the immune system and even the brain–gut–bone axis. Recently, probiotic-based dietary supplementation has emerged as an emerging strategy to improve bone health in chickens by regulating bone metabolism based on the gut–bone axis. This review aims to summarize the regulatory mechanisms of probiotics in the gut microbiota on bone metabolism and to provide new insights for the prevention and treatment of bone diseases in broiler chickens.

## 1. Introduction

Bone diseases lead to poor leg health, which is one of the common problems in poultry growth. Leg diseases affect poultry athletic ability, causing lameness and paralysis, and limiting further improvement in poultry performance and the quality of related meat products [1,2]. The improvement of intensive farming and management technology has promoted the muscle growth rate of broilers [3]. However, excessive muscle growth in broilers results in the inability of broiler leg bones to support body weight, and the problem of leg diseases is becoming increasingly prominent [4]. It is estimated that approximately 12.5 billion poultry worldwide suffer from various leg injuries each year [5]. There are several common bone diseases in the poultry industry, including tibial dyschondroplasia (TD), valgus–varus deformity (VVD), femoral head necrosis (FHN), and bacterial chondronecrosis with osteomyelitis (BCO) [2,3,6,7,8]. In the UK, around 27% of morbid broilers have dyskinesia, and 3.3% of them cannot move [9]. TD is a nutritional metabolic disease that occurs mainly in fast-growing broilers and is closely related to many influencing factors, such as growth rate, living environment, diet composition, etc., [4,10,11,12]. The prevalence of TD is over 10% in China [13], and its clinical features mainly manifest as the inability to stand or stand on one wing, which affects food and water intake and reduces growth performance [1,10]. Additionally, VVD causes several problems in the broiler, such as difficulty walking and lameness. The incidence of VVD in broilers varies at different ages. Cruickshank and Sim have shown that the incidence of the disease at 5 weeks of age was 5.4%; however, the incidence at the first week of age was only 0.4% [14]. BCO usually occurs in chickens between 14 and 70 days of age [15]. Therefore, leg health is an integral part of the broiler production economy. These leg diseases have seriously affected the exercise ability of broilers and have caused huge losses to the poultry industry.

The reasons for the occurrence of leg diseases in broilers are complex, including genetics, nutrition, feeding management, various infectious diseases, and other influencing factors [16,17,18,19,20]. Various approaches have been found to prevent and treat leg diseases in broilers. Several studies found that long-term breeding for leg health can reduce the incidence of leg diseases, although it has a certain impact on body weight [3,16,21]. People provide a suitable breeding environment for broilers by strengthening the regulation of environmental indicators such as temperature, air quality, lighting program, stock density, and litter, which is an important measure to alleviate the occurrence and development of broiler leg diseases [19,22]. However, the prevention of leg diseases in broiler production by regulating environmental indicators is a long-term process which will increase production costs. In addition, medication is an important measure to prevent and treat leg disease in broilers. Bone disease caused by various infectious factors, such as bacteria, can be treated and prevented with vaccines and antibiotics [8,15,17]. Nevertheless, the abuse of antibiotics can also promote bone disease.

**Figure 1:**
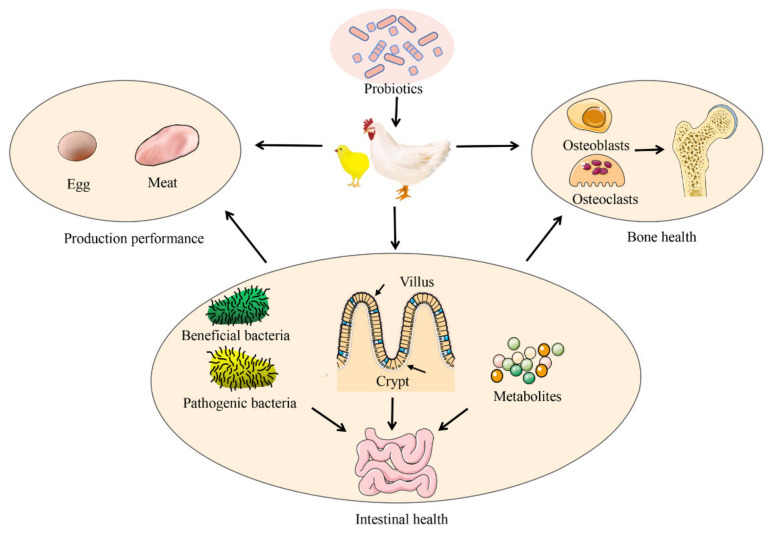
Application of probiotics in broiler production.

The variation in the gut microbiota caused by antibiotics, pesticides, and other factors may reduce intestinal barrier function, thus affecting bone health [12,23,24]. The difference in gut microbiota between TD and healthy chickens was revealed using a high-throughput sequencing technology [25]. Studies have shown that microbial homeostasis plays an essential role in bone diseases, and it is an effective method to prevent the occurrence of bone diseases in broilers by selecting various feed additives to regulate the gut microbiota [26,27,28]. Supplementation with probiotics can restore the composition of the gut microbiota and introduce beneficial functions into the gut microbiota, thereby improving or preventing disease. More importantly, probiotics have the advantages of safety, convenience, and natural nontoxicity; as a result, researchers have been flocking to this area in recent years. Previous studies have mainly focused on the effects of probiotics on poultry production performance, egg, and meat quality, intestinal health, and anti-stress phenomena (Figure 1) [29,30]. Recent studies have confirmed that probiotics also play an important role in maintaining bone health [17,24,28,31,32]. Supplementation with probiotics can regulate intestinal microbiota homeostasis and has broad application prospects in the prevention and treatment of bone disease [24,33,34]. This review illuminates the mechanism of the probiotics that regulate bone metabolism through the gut microbiota to provide a theoretical basis for bone-related studies in broiler chickens.

## 2. Probiotics and Gut Microbiota

### 2.1. Gut Microbiota

The animal gastrointestinal tract (GIT) is colonized by complex microbiota and is closely linked to microbiota homeostasis. The gut microbiota is the general term for the microbial community that inhabits the GIT of humans and animals, developing the synergetic effects associated with the host. The gut microbiota is derived mostly from the mother and then changes due to the influence of diet, geographical conditions, antibiotics, and other influencing factors; however, the composition of the gut microbiota is relatively stable in adulthood [35,36]. Some differences in the gut microbiota may exist between species. The diversity of the gut microbiota of poultry is low compared to that of other animals, which can be attributed to the rapid transport of food in the digestive system [37,38]. Furthermore, different parts of the intestine have different microbiota. The bacteria in the GIT of broilers are classified into fifteen phyla, although five main phyla (Firmicutes, Bacteroidetes, Proteobacteria, Actinobacteria, and Cyanobacteria) account for >90% of all sequences [38,39]. There were differences in the abundance of flora of the five intestinal segments, including the duodenum, jejunum, ileum, cecum, and colon. The sequence reads of Firmicutes obtained from the small intestine and colon of broilers were significantly higher than those in the cecum [39]. Bacteroidetes was the dominant phylum in the cecum (>50%); however, it accounts for less than 10% in the other four intestine segments of broilers [39,40]. The Proteobacteria in the duodenum is more than in the other four intestine segments [39,40]. The main bacterial genera in all intestinal segments were *Lactobacillus* spp, *Enterococcus* spp, and *Bacteroides* spp [39,40,41,42].

The diversity of the gut microbiota and the health of the animal body influence and shape each other, and the gut microbiota maintains a relatively balanced state with the host under the steady state regulation of the external environment. The gut microbiota plays an important regulatory role in nutrition exchange and immune protection [43]. As an important part of the digestive process, the gut microflora can directly or indirectly provide important nutrients for broilers, including short-chain fatty acids (SCFAs), amino acids, and vitamins [44]. SCFAs are produced by the gut microbiota fermenting dietary fiber, including acetate, propionate, and butyrate. The inner surface of the broiler intestine was covered with a mucus layer composed of mucins, which was the barrier against bacteria penetrating the intestinal epithelium [24]. SCFAs are not only an energy and carbon source for broilers, but also can stimulate the proliferation of intestinal cells and regulate the production of mucins [45,46]. Similarly, the gut microbiota itself can be used as amino acids and vitamin suppliers to their host [47,48]. Furthermore, the gut microbiota can act as a virtual endocrine organ capable of producing a variety of compounds that modulate the activity of distant organs, including the brain [49]. The gut microbiota plays an undeniably key role in the occurrence and development of various diseases, including Type 2 diabetes mellitus [50], obesity [51], neurological diseases [52], and bone metabolic diseases [53]. Overall, it may be an effective mean for treatment or prevention with these diseases by targeting the regulation of the gut microbiota.

### 2.2. Probiotics Regulate Gut Microbiota Homeostasis

Probiotics are currently defined as ‘live microorganisms that when administered in adequate amounts confer a health effect on the host’ by the Food and Agriculture Organization of the United Nations and the WHO (FAO/WHO) [54]. Probiotics are non-pathogenic bacteria that can colonize and multiply in the host’s GIT while inhibiting or killing pathogenic bacteria [43]. Probiotics have promotional functions for the digestion and absorption of food and regulate immunity. Studies have shown that probiotics have a positive impact on microbial populations, digestive function, nutrient absorption, antioxidant capacity, and immune response in broilers [24,55,56,57,58]. Probiotics improve food digestion by stimulating the host to increase the secretion of digestive enzymes or by producing hydrolytic enzymes (phytase, lipase, amylase, and protease) that promote nutrient absorption [59,60]. In addition, probiotics can also promote the absorption of nutrients in broilers by restoring intestinal structures such as villi height and crypt depth and by increasing the abundance of beneficial bacteria in the intestine [24,30,31,58,61].

Interactions between the microbiota and the host are essential for host growth, development, and health. Detrimental alterations in the composition of the microbiota, such as an increase in the augmentation of harmful bacteria in the intestinal tract, can disrupt interactions and may lead to disease. Probiotics interfere with the growth of pathogenic bacteria through adhesion, rejection, nutrient competition, space occupation, and other behaviors, thereby regulating the balance of the gut microbiota and maintaining normal immune, nutritional, and digestive functions [29,33,43]. Probiotics compete with colonized pathogenic bacteria and can reduce their adhesion and colonization in the GIT. Supplementation of the diet with Lavipan (a multiprobiotic probiotic product) can reduce the invasion of *Campylobacter* spp in the GIT of commercial farm-raised broilers [62]. Diets supplemented with *Clostridium* (*C.*) *butyricum* or *Enterococcus* (*E.*) *faecium* regulate the distribution of broiler cecal microflora by inhibiting the growth of pathogenic bacteria such as *Escherichia coli* and *C. perfringens* and by promoting the growth of *Lactobacillus* spp and *Bifidobacterium* spp [55,56]. Likewise, the dietary supplementation of *E. faecium* to broilers increased the relative abundance of SCFA-producing bacteria and improved bone formation [57]. Probiotics can protect broilers from pathogenic bacteria by producing different metabolites such as hydrogen peroxide, bacteriocins, and organic acids [37,63,64]. *Lactobacillus* (*L.*) *salivarus* and *L. crispatus* may inhibit the colonization of pathogenic bacteria through the production of hydrogen peroxide [37,63]. *C. butyricus* can produce a bacteriocin that inhibits the growth of *C. difficile* in the GIT [64]. Additionally, this probiotic can produce volatile fatty acids, which inhibit pathogenic bacterial growth by regulating intestinal pH and promoting the growth of beneficial bacteria [55,65]. 

## 3. Gut Microbiota and Bone Metabolism

Bone is a highly mineralized organ that constantly undergoes metabolic renewal and stores minerals, such as calcium (Ca) and phosphorus (P), and maintains the mineralization balance and structural integrity. The osteoblast is responsible for bone formation, and osteoclast is responsible for bone resorption and commonly maintains a normal balance between bone mass and bone density. The study by Sjögren et al. has indicated that bone mineral density is regulated by the gut microbiota [66]. Germ-free (GF) mice displayed increased trabecular bone volume/tissue volume (BV/TV), an augmented trabecular number, and reduced osteoclast numbers compared to conventionally raised mice, indicating an impaired osteoclast in sterile mice resulting in decreased bone resorption. However, the gut microbiota of 3-week-old GF mice was reconstituted, and bone mass and the osteoclasts number were restored to normal, indicating that the gut microbiota was involved in bone metabolism [66].

**Figure 2:**
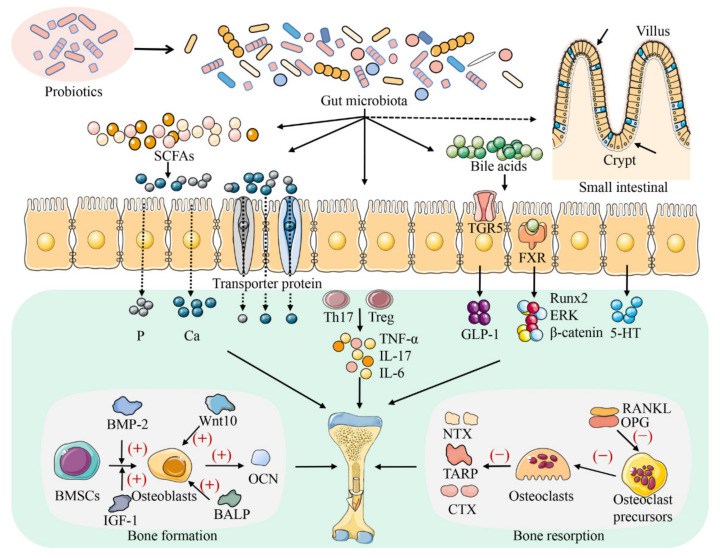
The regulatory mechanism of probiotics on bone metabolism. Probiotics affect the growth and development of bone by regulating the composition and function of the gut microbiota. Probiotics can maintain bone homeostasis by influencing intestinal nutrients absorption (Ca and P), metabolite production (SCFAs and bile acids), the balance of Th17-Treg cells, the secretion of downstream factors (TNF-α, IL-17, and IL-6), and the secretion of 5-HT in the ‘brain–gut–bone’ axis. Probiotics affect the absorption of nutrients in the intestinal tract mainly by regulating the renewal rate of intestinal epithelial cells and the mode of calcium and phosphorus transport. Furthermore, probiotics also affect bone growth and development by affecting the activity of osteoblastic markers (such as Wnt10, BMP-2, BALP, and OCN) and osteoclastic markers (such as OPG, RANKL, TRAP, NTX, and CTX).

Similarly, a close relationship between bone health and gut microbiota was also found in broilers. The abundance of microbiota in the small and large intestines of TD broilers was significantly different from that in healthy broilers examined using high-throughput sequencing [25,67]. Additionally, by analysing the intestinal microbial diversity of patients with osteoporosis and osteopenia, it was found that the bacterial composition and diversity of the two groups changed compared to that of the normal group [68]. Therefore, the gut microbiota is expected to provide a new alternative for the prevention and treatment of broiler bone diseases. Several studies have shown that changes in the gut microbiota affect the mechanical properties of bone tissue and affect bone tissue strength in mice [26,32,34,69]. Therefore, the diversity and composition of the gut microbiota have an impact on bone health (Figure 2). The types of bone diseases have differences between species; thus, the gut microbiota may play a different role in each. Regulation of the gut microbiota homeostasis in different species is important for the prevention and treatment of bone diseases [24,34,51,69]. 

### 3.1. Gut Microbiota Regulates Bone Metabolism by Improving Intestine Structure

The intestine tract is an important place for the body to obtain nutrients, and the intestinal mucosal structure, including the intestinal villi and crypts, is the physiological basis for the digestion and absorption of nutrients. The intestinal crypt is a tubular structure formed by the invagination of the epithelium at the base of the intestinal villi into the lamina propria. The intestinal epithelium cells are constantly migrating and differentiating from the base of the crypt to the end of the villi, forming villi cells with an absorptive capacity [30,46,61]. If the rate of the renewal of intestinal epithelial cells is inhibited, the depth of the crypt becomes shallow [61]. The length of the villus and the depth of the crypt are important parameters for evaluating the small intestinal function in broilers [30,61]. Increased villi length provides greater intestinal surface area and enhanced nutrition absorption, thus improving broiler growth and production [58]. Supplementing broiler diets with direct-fed microbial (DFM) extends and expands villus length and width, thus enhancing the intestinal absorption of nutrients [58,70]. A study by Zhao et al. has also demonstrated that *Bacillus* (*B.*) *licheniformis* H2 in feed could increase the ratio of villus height to crypt depth in broiler chicken, as well as improve the rate of nutrient absorption by the intestinal tract [61]. In addition to changing the physiological structures, such as intestinal villi and crypts, probiotics can also affect the absorption of nutrients related to bone development in broilers through the expression of shadow transporter proteins [57,71]. Ca and P are critical nutrient elements for bone growth in broilers. These minerals are deposited in bone tissue as hydroxyapatite under certain conditions, which constitutes the fundamental component of bone tissue, and are essential for maintaining the development, growth, and metabolism of bone in animals [72,73]. A study reported that the probiotic *Bifidobacterium longum* increases bone mineral density (BMD) by stimulating the absorption of minerals, such as Ca and P [74]. However, the gut microbiota can affect the intestinal absorption of Ca and P in various ways.

An in vitro study has reported that probiotics can affect the intestinal absorption of Ca by regulating transcellular and paracellular pathways [71]. For example, *L. plantarum* strains regulate the transcellular pathway by regulating the expression of vitamin D receptors and Ca transporters, while *L. delbrueckii* strains act on the paracellular pathway by regulating the expression of claudin-2 [71]. The duodenum was the main site for P absorption in the small intestine of broilers, and the absorption of P in this intestinal segment was mediated by a saturated carrier [75]. Type IIb sodium–phosphate cotransporter (NaP-IIb) is an important transporter of P in the small intestine and is mainly expressed in the duodenum of broilers. Wang et al. found that *E. faecium* could improve the P utilization rate of broilers by regulating the gut microbiota and up-regulating NaP-IIb mRNA expression in the small intestine [57]. Together, the gut microbiota exerts a protective effect on bone by regulating intestinal structure.

### 3.2. Gut Microbiota Regulates Bone Metabolism through the Host Metabolic System

The gut microbiota can play an essential role in bone homeostasis by regulating the production of SCFAs and other metabolites (Figure 2) [57,76]. SCFAs are organic fatty acids with two to six carbon atoms, mainly including acetic acid, propionic acid, butyric acid, etc., [77]. SCFAs are metabolites produced by the interaction between diet and gut microbiota and are considered to be a key link between gut microbiota and bone [44,77]. Gut microbiota can promote the intestinal absorption of nutrients by regulating the production of SCFAs. Supplementation with *E. faecium* can change the gut microbiota in broilers, increasing the relative abundance of SCFA-producing bacteria, and improving intestinal P absorption, bone formation, and metabolic activities [57]. The protective effect of SCFAs on bone is related to microbial fermentation in the colon and SCFAs’ production [78]. SCFAs can create an acidic environment by reducing the pH value of the intestinal tract, thereby increasing the solubility of Ca or other mineral elements and promoting mineral absorption and bone mineralization [79]. In addition, insulin-like growth factor 1 (IGF-1) is a hormone that acts on bone growth and development by promoting osteoclast differentiation [80]. Previous research has revealed that SCFAs induce the production of IGF-1 in the liver and adipose tissue to regulate bone health [81]. In addition to affecting mineral absorption, SCFAs (acetate, propionate, and butyrate) can directly or indirectly affect bone health by influencing bone metabolism-related cells such as osteoclasts and osteoblasts [77]. Propionate and butyrate can alter the metabolic state of preosteoclasts, thus preventing osteoclast differentiation [82]. A study has shown that butyrate promotes osteoblast production by activating the Wnt signaling pathway in osteoblasts [83]. Therefore, the role of SCFAs in bone homeostasis is difficult to ignore.

Bile acids are the main organic components of bile and can be divided into primary and secondary bile acids according to their sources. Primary bile acids are synthesized directly from cholesterol in liver cells [84]. During intestinal transport, conjugated primary bile acids undergo uncoupling and dehydrogenation reactions in the gut microbiota, leading to the formation of secondary bile acids, such as deoxycholic acid and lithic bile acids [84,85]. Bile acids may play a vital role in bone metabolism. A study on postmenopausal women showed that the serum levels of bile acids were positively correlated with BMD and negatively correlated with the bone turnover biomarkers of bone resorption [86]. Gut microbiota alters the amount and type of secondary bile acids through farnesoid X receptor (FXR) and G protein-coupled bile acid receptor 5 (TGR5) signaling, resulting in different metabolic effects [87]. FXR acts as a bile acid sensor to control bile acid homeostasis and plays an important role in bone metabolism [88]. In vitro assays revealed that bile acids can regulate bone metabolism by activating FXR signaling and upregulating Runt-related transcription factor 2 (Runx2) expression, and by enhancing extracellular signal-regulated kinase (ERK) and β-catenin signaling [88]. Glucagon-like peptide-1 (GLP-1), an intestinal hormone, activates the proliferation of thyroid C cells and promotes the secretion of calcitonin, thereby inhibiting bone resorption while stimulating the proliferation of osteoblasts to promote bone formation [87,89]. Secondary bile acids are agonists of TGR5 and indirectly regulate the process of bone metabolism by activating TGR5 to increase the production of GLP-1 [89]. In addition, osteoporosis can be reduced by increasing the key microbiota that drives changes in amino acids and fatty acids [90]. However, there is little information on the regulation of skeletal development in broiler chickens by the gut microbiota through host metabolism, for which in-depth studies are warranted.

### 3.3. Gut Microbiota Modulates Bone Metabolism via Immune and Inflammatory Factors 

As the largest immune organ in animals, the intestine is an important line of defense for the body against invasion by pathogenic microorganisms. Correspondingly, the gut microbiota plays a vital role in the regulation of bone metabolism through immune and inflammatory factors (Figure 2) [29,91,92]. The microbiota in the gut is closely associated with Treg cells and helper T (Th) cells [66,87]. Treg cells have been shown to influence bone resorption by impeding the differentiation of monocytes into osteoclasts [93]. Th17 cells are known subsets of osteogenic T cells in the CD4 T cell lineage and promote osteoclastogenesis via the production of the receptor activator of nuclear factor kappa B ligand (RANKL), tumor necrosis factor-alpha (TNF-α), interleukin (IL)-6, and IL-17 [94,95]. Restoring gut microbiota homeostasis is important in improving bone health [34,96]. Previous research has indicated that probiotics could prevent alveolar bone loss by rebuilding the gut microbiota to restore the intestinal barrier and bone marrow Th17/Treg [34]. Britton et al. found that *L. reuteri* supplementation in ovariectomized (OVX) mice counteracted bone loss by suppressing the level of CD4^+^ T lymphocytes in the bone marrow and the expression of IL-6, tartrate-resistant acid phosphatase (TRAP), and RANKL [96]. Similarly, this process involves the restoration of gut microbiota homeostasis. TNF-α is an inflammatory cytokine that inhibits the differentiation of mesenchymal cells into osteoblasts and mediates the formation of osteoclasts, thereby inhibiting the process of bone formation and participating in the occurrence of inflammatory bone diseases such as rheumatoid arthritis (RA) [97,98,99]. Disruption of the gut microbiota activates the expression of T cells, leading to enhanced TNF-α expression in the bone marrow [95,96]. However, supplementation with some beneficial bacteria can alleviate bone diseases by decreasing the expression of inflammatory factors [95,100,101]. In a study on arthritic rats, the oral administration of *L. acidophilus* was found to decrease levels of pro-inflammatory factors (TNF-α and IL-6) and increase levels of anti-inflammatory factors (IL-10) to alleviate arthritis [100]. The addition of *L. acidophilus* to the broiler diet also reduced the expression of IL-6 and TNF-α [101]. Sjögren et al. found that, compared to conventionally raised mice, GF mice displayed reduced levels of CD4^+^ T cells (promoting osteoclastogenesis), TNF-α, and osteoclast precursor cells [66]. However, this phenomenon improved when the normal microbial community was colonized for GF mice of 3 weeks age [66]. These findings suggest that gut microbiota-mediated immunity and inflammation play a critical role in bone physiology.

### 3.4. Gut Microbiota Regulates Bone Metabolism through the Brain–Gut–Bone Axis

Sensory neurons, immune mediators, gut hormones, and other molecules derived from the gut microbiota transmit information between the gut and the brain, also known as the ‘gut–brain’ axis [102]. 5-hydroxytryptamine (5-HT) is an essential neurotransmitter in the ‘brain–gut’ axis [49,76]. Tryptophan hydroxylase (TPH) catalysis is the rate-limiting step in 5-HT synthesis, and the expression of this enzyme has become a marker of 5-HT synthesis [103]. 5-HT is produced both peripherally and centrally, the former mainly through intestinal enterochromaffin cells (ECs) in the GIT using TPH1 and the latter mainly through the brain using TPH2 [103,104]. The changes in 5-HT levels from two different sources do not interfere with each other, probably because 5-HT cannot cross the blood–brain barrier. Previous studies have found that 5-HT receptors are identified in osteoblasts [105,106,107,108]. Different sources of 5-HT have different effects on bone mass [108]. Brain-derived 5-HT, a neurotransmitter, can lead to increased bone formation and decreased bone resorption by inhibiting sympathetic nerve activity [107]. Conversely, 5-HT derived from the gut acts as a hormone to hinder bone formation [108]. 5-Hydroxytryptamine receptor 1b (Htr1b) is the receptor responsible for the effect of serotonin on osteoblasts [106]. In osteoblasts, the binding of enteric-derived 5-HT to Htr1b inhibited the production of cyclic adenosine 3′,5′-monophosphate (cAMP) while inhibiting protein kinase A (PKA)-mediated cAMP response element binding (CREB) phosphorylation [106,108]. This process inhibits the proliferation of osteoblasts [106,108]. Inhibition of gut-derived 5-HT in OVX rodents was found to prevent bone loss and inhibit the development of osteoporosis [109]. Conventionally raised mice presented increased 5-HT levels and decreased trabecular BV/TV compared to GF mice [66]. These results suggest that the gut microbiota affects bone development by regulating the expression of 5-HT. SCFAs are a metabolite of the intestinal microbiota that can affect bone development by acting on ECs and by promoting TPH1 expression and 5-HT production [110]. *B. subtilis*-based dietary supplementation has been reported to improve bone traits in broilers possibly by increasing the intestinal absorption of Ca and decreasing bone resorption through the inhibition of sympathetic nerve activity by the central serotonergic system [111]. Thus, the brain–gut–bone axis occupies an important position in the regulation of bone health by the gut microbiota.

## 4. Probiotics Regulates Bone Metabolism

### 4.1. Bone Formation

Osteoblasts are responsible for bone formation. Probiotics act on osteoblasts in multiple ways and are involved in bone homeostasis (Figure 2 and Table 1). *L. reuteri* affects osteoblastic activity by stimulating cytokine secretion in the T cells of mesenteric lymph nodes [112]. Wnt10b is a bone anabolic Wnt ligand and has the ability to enhance osteogenesis and angiogenesis to promote skeletal bone defect healing [113]. The study by Tyagi et al. found that LGG supplementation promoted bone formation by increasing the number of Treg cells in the bone marrow, the butyrate level in the gut, and by activating Wnt signaling in osteoblasts in mice [83]. Bone morphogenetic proteins (BMPs) and members of the transforming growth factor-β (TGF-β) superfamily regulate bone homeostasis by balancing anabolic and catabolic activities between osteoblasts and osteoclasts. BMP-2 is the main cytokine that promotes bone formation, which can regulate the differentiation of bone marrow mesenchymal stem cells (BMSCs) to osteoblasts, inhibiting the apoptosis of cells [114,115,116]. A study by Parvaneh et al. confirmed that supplementation of *Bifidobacterium longum* in OVX rats resulted in an increase in femoral BMD associated with the high expression of BMP-2 [117].

Osteoprotegerin (OPG), bone-specific alkaline phosphatase (BALP), and osteocalcin (OCN) are critical markers in bone formation and are often used as important indicators of bone health. OPG is mainly produced by osteoblasts or stromal cells, which can inhibit the formation and differentiation of osteoclasts and indirectly increase BMD. RANK exists on the surface of osteoclast precursors and osteoclasts and is a receptor for RANKL. The OPG/RANKL/RANK axis is a crucial signaling pathway in bone metabolism. OPG can bind to RANKL during osteoclast differentiation by inhibiting the binding of RANKL and RANK, blocking the differentiation of osteoclast precursors into osteoclasts [118]. Yeom et al. indicated that *Propionibacterium(P.) freudenreichii* enhanced bone mineralization by increasing the OPG/RANKL ratio and the level of BMP-2 [119]. BALP, an indicator of osteoblast proliferation or bone remodeling, typically increases bone formation during the growth phase [120]. Supplementation with probiotics in postmenopausal women with osteopenia has shown that probiotics have beneficial effects on bone by reducing serum BALP levels and by slowing the rate of bone turnover [121]. OCN is produced by osteoblasts and is the abundant non-collagenous protein in bone. Probiotics were administered in male mice, and serum OCN levels and bone formation rates significantly increased [122]. LGG prevents Tenofovir disoproxil fumarate (TDF)-induced mandibular bone loss in mice by up-regulating OCN expression and the proliferation and osteogenesis of mesenchymal stem cells [123]. Furthermore, a study by Guo et al. indicated that *B. subtilis* PB6 can increase the expression of OCN and BALP in broiler chickens, regulating tibial development [124]. These studies have shown that probiotics can promote bone growth and development in various ways.

### 4.2. Bone Resorption 

Probiotics can affect bone metabolism by regulating bone resorption, which has a few biochemical markers, such as the TRAP, N-terminal peptide (NTX), and C-terminal peptide (CTX) of collagen type I (COLI) (Figure 2 and Table 1). TRAP is mainly expressed in mature osteoclasts and is less abundant in osteoblasts. TRAP is a key marker in osteoclast differentiation, and TRAP-positive multinucleated cells (≥3 nuclei) are considered as osteoclasts [129]. TRAP-5b, a subtype of TRAP, is an osteoclast-specific enzyme that reflects the number of osteoclasts [130]. Previous studies have found that *P. freudenreichii*, *L. reuteri*, and LGG can reduce the formation of osteoclasts using TRAP staining [95,96,126]. Degradation products of COLI, such as CTX and NTX, are produced during active bone resorption and can be used as surrogate markers of osteoclast function [130]. Takimoto et al. found that TRAP-5b and urinary NTX levels were reduced in postmenopausal women after treatment with *B. subtilis*, indicating that *B. subtilis* can improve BMD by inhibiting the number and the activity of osteoclasts [127]. Serum CTX is produced by osteoclasts and is a marker of bone resorption. *Bifidobacterium longum* inhibits bone resorption by reducing serum CTX levels in OVX rats [117]. Similar results were observed when 1-day-old broilers were fed with a mixed diet with *B. subtilis* (1.0 × 10^6^ spores/g feed) [111]. When broilers reached 43 days of age, probiotic-fed broilers had a greater tibial and femoral lateral wall thickness than basal-fed broilers, which may be associated with reduced serum CTX levels [111]. 

In addition, probiotics also affect bone resorption by modulating immune cells and cytokines. Treatment with *L. reuteri* in OVX mice inhibited the bone marrow CD4^+^ T lymphocytes osteoclast-mediated bone resorption in vitro, preventing bone loss [109]. Lactobacillus strains effectively improve RA by modulating T-cell responses and restoring the gut microbiota balance [129,131]. Cytokines produced during the immune response are closely related to bone metabolism, among which RANKL, TNF-α, and IL-17 can regulate osteoclastogenesis [95,132]. RANKL is a crucial cytokine during osteoclast differentiation and its mastered bone resorption. *L. rhamnosus* attenuates bone loss in OVX mice by regulating Treg-Th17 cell balance, leading to an inhibition of RANKL-induced osteoclastogenesis [125]. *P. freudenreichii* improves collagen-induced arthritis by inhibiting RANKL-induced osteoclast differentiation through the nuclear factor kappa-B (NF-κB) signaling pathway [126]. TNF-α plays a central role in osteoclastogenesis and can enhance RANKL signaling [98]. IL-17 can promote the formation of osteoclasts and stimulate the expression of RANKL and TNF-α. Some studies have shown that IL-17 is involved in the development of postmenopausal osteoporosis, inflammatory arthritis, and alveolar bone loss [34,133,134]. LGG inhibits TDF-induced osteoclastogenesis in mice by down-regulating the expression of RANKL, TNF-α, and IL-17 in the bone marrow [95]. Yan et al. found that *B. subtilis* can improve bone development in broilers by inhibiting inflammation at high ambient temperature, such as by reducing the TNF-α level [111]. Similarly, this variation has been found in laying hens. *B. subtilis* can be used as a dietary supplement to protect the bone health of laying hens by inhibiting gut and systemic pro-inflammatory factors and by increasing the OPG/RANKL ratio [128]. These results suggest that probiotics can regulate bone growth and development, and that immune response is also involved in probiotic-mediated bone resorption. While, the regulatory mechanisms of probiotics are cross-linked with bone metabolism, this needs to be further explored.

## 5. Application of Probiotics 

Probiotics affect bone metabolism by regulating the composition and function of the gut microbiota (Table 1). Bacteria of the genera *Bacillus* and *Lactobacillus* have been used as probiotics and are widely used in animal feeding. *Bacillus* spores can survive in harsh environments, such as high temperature, high pressure, and extreme pH, and can be used as a commercial animal feed additive to maintain animal health [70,135]. Supplementation with *Bacillus*-DFM was found to help maintain gut microbiota balance in broilers and significantly increase tibial fracture strength and bone mineralization [70]. In addition, *B. subtilis*, *B. licheniformis*, and *B. cereus* are commonly used commercial probiotic strains of *Bacillus* which promote the development of the tibia [31,136,137]. Broilers were fed with feed containing *B. subtilis* to promote tibial development and gut microbiota balance, and this increased the total lactic acid bacteria count [28,70]. Additionally, the Sadeghi’s study found that *B. subtilis* ameliorates the reduction of tibial bone mass in chicks caused by *Salmonella enteritidis* infection [138]. Therefore, supplementation with *B. subtilis*-based probiotics is conducive to broiler bone health. 

The bacteria of the genus *Lactobacillus* are important probiotics, including *L. reuteri*, *L. rhamnosus*, and *L. acidophilus*, and they affect BMD in animals and humans. Supplementation with *L. reuteri* 6475 can reduce bone loss in OVX mice by increasing BMD in the regulation of T cells in healthy male mice [96,112,122]. *Lactobacillus rhamnosus* GG (LGG) affects the composition of the gut microbiota and the mechanical properties of long bones in immunodeficient mice [139]. In addition, Tyagi et al. indicated that LGG increased bone mass in mice by stimulating butyrate production in the gut microbiota [83]. Similarly, *L. rhamnosus* can also affect the development of broiler bone. A study has demonstrated that *L. rhamnosus* JYLR-005 prevented thiram-induced TD by maintaining the morphological structure of the chondrocytes and by improving broiler bone-related growth performance, including the weight, length, and mean diameter of the tibia [32]. *L. acidophilus* inhibited bone loss by regulating Treg-Th17 cell balance and increasing BMD, bone heterogeneity, and femoral and tibial microarchitecture in OVX mice [140]. Moreover, the contents of Ca and P in the tibia of broilers were increased after using a feed supplement with a combination of *L. acidophilus* and *L. plantarum*, which promoted tibial development [141]. Therefore, supplementation with appropriate probiotics in animal feeding can effectively maintain bone health.

## 6. Conclusions

Probiotics are widely used in animal feeding as a dietary supplement to maintain health. In fact, probiotics and gut microbiota influence the dynamic balance of bone formation and bone resorption through several pathways in different animal species. However, more explicit mechanisms are needed to further investigate the relationship between probiotics and gut microbiota in broiler bone health. Correspondingly, probiotics regulating the activity of osteoblasts and/or osteoclasts are more conducive to target the treatment and/or prevention of bone diseases in broilers, and provide a theoretical basis for the further exploration of efficient breeding methods of broilers. However, more studies have focused on the effect of probiotics on the skeletal development of broiler chickens, and there is a lack of thorough investigations on the treatment of bone diseases by regulating gut microbiota using probiotics. Furthermore, the gut microbiota is only one factor in the regulation of bone metabolism, and there may be more factors including age, diet, and physical condition involved in this process. Therefore, all these factors need to be further considered in depth by subsequent studies.

## Figures and Tables

**Table 1:** Effect of probiotics on bone metabolism.

Function	Bacterial Strain	Species	Experimental Cycle	Administration Route	Effect	References
Promoting bone formation	*L. reuteri*	Mice	4 weeks	oral gavage	Stimulate T cells in mesenteric lymph nodes to increase cytokine secretion	[96,112,122]
	*L. rhamnosus*	Mice	4 weeks	oral gavage	Increase the level of butyrate in the gut, elevate the number of Treg cells in the bone marrow, and up-regulate the expression of Wnt10b and OCN	[83]
	*L. rhamnosus* JYLR-005	Broiler chickens	15 days	dietary supplement	Increase the content of Ca and P in serum and bone	[32]
	*P. freudenreichi*	Rats	4 months	oral gavage	Increase the OPG/RANKL ratio and BMP-2 levels	[119]
	*B. subtilis* and *B. amyloliquefaciens*	Broiler chickens	28 days	dietary supplement	Increase the contents of Ca and P in bone	[70]
	*B. subtilis*	Broiler chickens	42 days	dietary supplement	Increase the expression of OCN and BALP and increase the concentration of P in the tibia	[28,124]
	*E. faecium*	Broiler chickens	42 days	dietary supplement	Up-regulate the mRNA expression of NaP-IIb and increase the P of bone	[57]
Suppressing bone resorption	*L. reuteri*	Mice	4 weeks	drink water	Inhibit the proliferation of CD4^+^ T lymphocytes in the bone marrow and reduce osteoclastogenesis	[96]
	*L. rhamnosus*	Mice	45 days	oral gavage	Regulate the balance of Treg-Th17 cells; inhibit the expression of RANKL, TNF-α, IL-17, and the formation of osteoclasts	[125]
	*P. freudenreichii*	Mice	56 days	oral gavage	Inhibition of RANKL-induced osteoclast differentiation through NF-κB signaling pathway	[126]
	*B. subtilis*	humans	24 weeks	dietary supplement	Inhibit the number and activity of osteoclasts by reducing the levels of TRAP-5b and urinary NTX	[127]
	*B. subtilis*	Broiler chickens	43 days	dietary supplement	Inhibit sympathetic nerve activity by the central serotonergic system and decrease the CTX content in serum	[111]
	*B. subtilis*	Laying Hens	61 days	dietary supplement	Inhibit the content of pro-inflammatory factor synthesis (e.g., IL-1 and TNF-α) and increase the OPG/RANKL ratio	[128]
	*Bifidobacterium longum*	Rats	16 weeks	oral gavage	Decrease the CTX content in serum	[117]
